# The Microbiome-Gut-Brain axis regulates social cognition & craving in young binge drinkers

**DOI:** 10.1016/j.ebiom.2023.104442

**Published:** 2023-02-02

**Authors:** Carina Carbia, Thomaz F.S. Bastiaanssen, Luigi Francesco Iannone, Rubén García-Cabrerizo, Serena Boscaini, Kirsten Berding, Conall R. Strain, Gerard Clarke, Catherine Stanton, Timothy G. Dinan, John F. Cryan

**Affiliations:** a APC Microbiome Ireland, University College Cork, Cork, Ireland; b Department of Health Sciences, University of Florence, Florence, Italy; c Teagasc Food Research Centre, Moorepark, Co. Cork, Ireland; d Department of Psychiatry and Neurobehavioural Science, University College Cork, Cork, Ireland; e Department of Anatomy and Neuroscience, University College Cork, Cork, Ireland

**Keywords:** Microbiome, Social cognition, Binge drinking, Adolescence, Gut-brain axis

## Abstract

**Background:**

Binge drinking is the consumption of an excessive amount of alcohol in a short period of time. This pattern of consumption is highly prevalent during the crucial developmental period of adolescence. Recently, the severity of alcohol use disorders (AUDs) has been linked with microbiome alterations suggesting a role for the gut microbiome in its development. Furthermore, a strong link has emerged too between microbiome composition and socio-emotional functioning across different disorders including AUD. The aim of this study was to investigate the potential link (and its predictive value) between alcohol-related altered microbial profile, social cognition, impulsivity and craving.

**Methods:**

Young people (N = 71) aged 18–25 reported their alcohol use and underwent a neuropsychological evaluation. Craving was measured at baseline and three months later. Diet was controlled for. Blood, saliva and hair samples were taken for inflammatory, kynurenine and cortisol analysis. Stool samples were provided for shotgun metagenomic sequencing and short-chain fatty acids (SCFAs) were measured.

**Findings:**

Binge drinking was associated with distinct microbiome alterations and emotional recognition difficulties. Associations were found for several microbiome species with emotional processing and impulsivity. Craving showed a strong link with alterations in microbiome composition and neuroactive potential over time.

**Interpretation:**

In conclusion, this research demonstrates alterations in the gut microbiome of young binge drinkers (BDs) and identifies early biomarkers of craving. Associations between emotional processing and microbiome composition further support the growing literature on the gut microbiome as a regulator of social cognition. These findings are of relevance for new gut-derived interventions directed at improving early alcohol-related alterations during the vulnerability period of adolescence.

**Funding:**

C.C. and R.G-C. received funding from the European Union's Horizon 2020 research and innovation programme under the Marie Sklodowska-Curie grant agreement No. 754535. APC Microbiome Ireland is a research centre funded by 10.13039/501100001602Science Foundation Ireland (SFI), through the Irish Government’s National Development Plan [grant no. SFI/12/RC/2273_P2]. J.F.C has research support from Cremo, 10.13039/100007082Pharmavite, 10.13039/100004352DuPont and Nutricia. He has spoken at meetings sponsored by food and pharmaceutical companies. G.C. has received honoraria from Janssen, Probi, and Apsen as an invited speaker; is in receipt of research funding from 10.13039/100007082Pharmavite, 10.13039/501100003144Fonterra, Nestle and Reckitt; and is a paid consultant for 10.13039/501100012030Yakult, Zentiva and Heel pharmaceuticals. All the authors declare no competing interests.

**Research in context**

**Evidence before this study**

Accumulating evidence suggests that alterations in the gut microbiome may play a role in alcohol use disorders (AUDs). Seminal research has demonstrated that AUD patients exhibit gut microbiome alterations which seem to be lined to severe craving. Performing a faecal matter transplant from alcoholic patients to mice the authors demonstrated that gut microbiome changes associated with alcohol were able to induce brain and behavioral disturbances including impaired sociability in mice. These observations have led to postulate a role of the gut microbiome in the development of AUD. However, little is known about alcohol-related changes in the gut microbiome and cognitive and psychological correlates in early stages of alcohol misuse. Examining the effects of binge drinking—the most common pattern of alcohol misuse during adolescence—could help identify early and novel biomarkers during this vulnerable period before an addiction develops.

**Added value of this study**

By focusing on adolescence, a crucial time of both brain and gut-immune development, we identify gut microbiome alterations linked to binge drinking in young people. The microbiome composition showed associations with social cognition and impulsivity, adding support to the growing literature on cognition-microbiome links. Alterations in the gut microbiome composition and the neuroactive potential were associated with higher craving over time, constituting interesting candidates for early biomarkers.

**Implications of the available evidence**

This study demonstrates that the most common pattern of alcohol misuse during adolescence is linked with gut microbiome alterations, even before an addiction develops. Furthermore, it highlights the importance of the gut microbiome in regulating craving and social cognition. These findings could help develop novel (dietary or pre/probiotic) interventions directed at improving early alcohol-related alterations in young drinkers during the vulnerable period of adolescence.

## Introduction

Binge drinking is the most common pattern of alcohol misuse during adolescence in Western countries. This pattern of repeated intoxications is usually defined as the consumption of a large amount of alcohol within a short period of time, leading to a blood alcohol concentration of at least 0.8 g/l.^1^ One in three young Europeans and North Americans engage in frequent binge drinking.^2^

Binge drinking peaks during late adolescence, which is considered to last up to 24 years of age,^3^ and has been associated with cognitive and anatomical alterations^4^, ^5^, ^6^ and increased risk of developing psychopathology and alcohol use disorder (AUD) later in life.^7^ The nature of the processes involved in the development of alcohol addiction are still not completely resolved but a role of the gut microbiome has been suggested.^8^^,^^9^ The microbiome-gut-brain axis conforms a bidirectional pathway of communication through the stimulation of cytokine expression, microbial metabolites such as short-chain fatty acids (SCFAs), tryptophan metabolism, cortisol, among others.^10^ Seminal research has demonstrated that patients with AUD that underwent detoxification present an increase in gut permeability, gut microbiome alterations, as well as higher levels of circulating cytokines and cortisol that were related to AUD severity and craving.^9^^,^^11^ Interestingly, gut microbiome alterations were associated with impaired sociability in chronic alcohol use. After transplantation of gut microbiome from AUD patients to mice, the authors demonstrated that this manipulation induced brain function disturbances such as neuroinflammation and behavioural alterations including impaired sociability.^8^ These observations have led to postulate a role of the gut microbiome in the development of AUD.

Interestingly, the gut microbiome appears to be intimately connected to social behaviour,^12^ including psychiatric disorders with persistent social cognition deficits.^13^, ^14^, ^15^, ^16^ Social cognition is still undergoing significant neurodevelopment during adolescence.^17^^,^^18^ Deficits in social cognition have been reported in adolescents who binge drink and are of relevance for drinking escalation and the development of future addiction.^19^

Emerging preclinical evidence examining the effects of binge drinking on the microbiome suggests that this pattern causes more subtle but significant changes in microbial composition and, importantly, some of the alterations observed during adolescence persisted into adulthood.^20^ Thus, developmental vulnerabilities to alcohol during adolescence might extend beyond the brain into the gut microbiota,^21^^,^^22^ which is likely to affect the adult host health.^23^ In fact, alterations in the microbiota-gut-brain axis could contribute to accelerate the cycle of addiction.^24^^,^^25^

This study is aimed at identifying early biomarkers of alcohol misuse by exploring the gut microbiome and neurocognitive correlates in young binge drinkers (BDs) in the absence of addiction. Our interest was to focus on adolescence, a crucial time of both brain and gut-immune development, and on healthy adolescents, with no alcohol use disorders, who displayed binge drinking—the most common pattern of alcohol misuse during this time. First, we examined the potential link between binge drinking, microbiome alterations and social cognition and impulsivity, which are cognitive domains repeatedly linked to drinking escalation. At the same time, social cognition and impulsivity have been both associated with microbiome disturbances across a number of disorders.^8^^,^^12^^,^^15^^,^^21^ Secondly, we aimed to identify early biomarkers of craving by examining associations between microbiome disruptions and craving over time. Additionally, we examined potential alterations in key mediators (i.e., tryptophan/kynurenic acid pathway metabolites and cortisol markers) for microbiome-gut-brain axis communication that are known to be implicated in social cognition.^21^^,^^26^^,^^27^

## Methods

### Study design

Young healthy people between 18 and 25 years of age living in Cork (Ireland) were recruited for the study. We followed the World Health Organisation (WHO) definition, that is, a binge drinking episode was defined as at least 60 g or more of pure alcohol on at least one occasion in the past 30 days, which leads to a blood alcohol concentration (BAC) of around 0.8 g/l or higher.^28^^,^^29^ A standard drink in Ireland has about 10 g of pure alcohol. First, participants underwent a clinical interview in which demographic, clinical data and information on confounders such as diet were recorded. Then, participants underwent a second visit in which a neuropsychological evaluation was performed and biological samples (saliva, hair, blood and stool) were collected. Participants were instructed to not drink alcohol the day before the second visit and to abstain from drinking more than 2 units two days before. Finally, a 3-month follow-up (mean = 106 days [SD = 11 days]) was performed in which participants reported their alcohol use and craving. All participants gave written informed consent and received up to 50 euros compensation, which was approved by the ethical committee and constitutes a common practice. No significant selection bias (i.e., certain population groups aren't covered in polling or survey sampling, leading to skewed sample data results) or response bias (i.e., respondent's desires to be socially accepted in the way they reply to questions) was expected from the economical compensation in the present study.

The study protocol was approved by the Clinical Research Ethics Committee of the Cork Teaching Hospitals (protocol number APC099) and conducted in accordance with Good Clinical Practice guidelines and the Declaration of Helsinki. Informed consent was obtained from all participants prior to enrolment into the study. This study was in compliance with General Data Protection Regulations and no third parties were involved.

### Participants

**Fig. 1:**
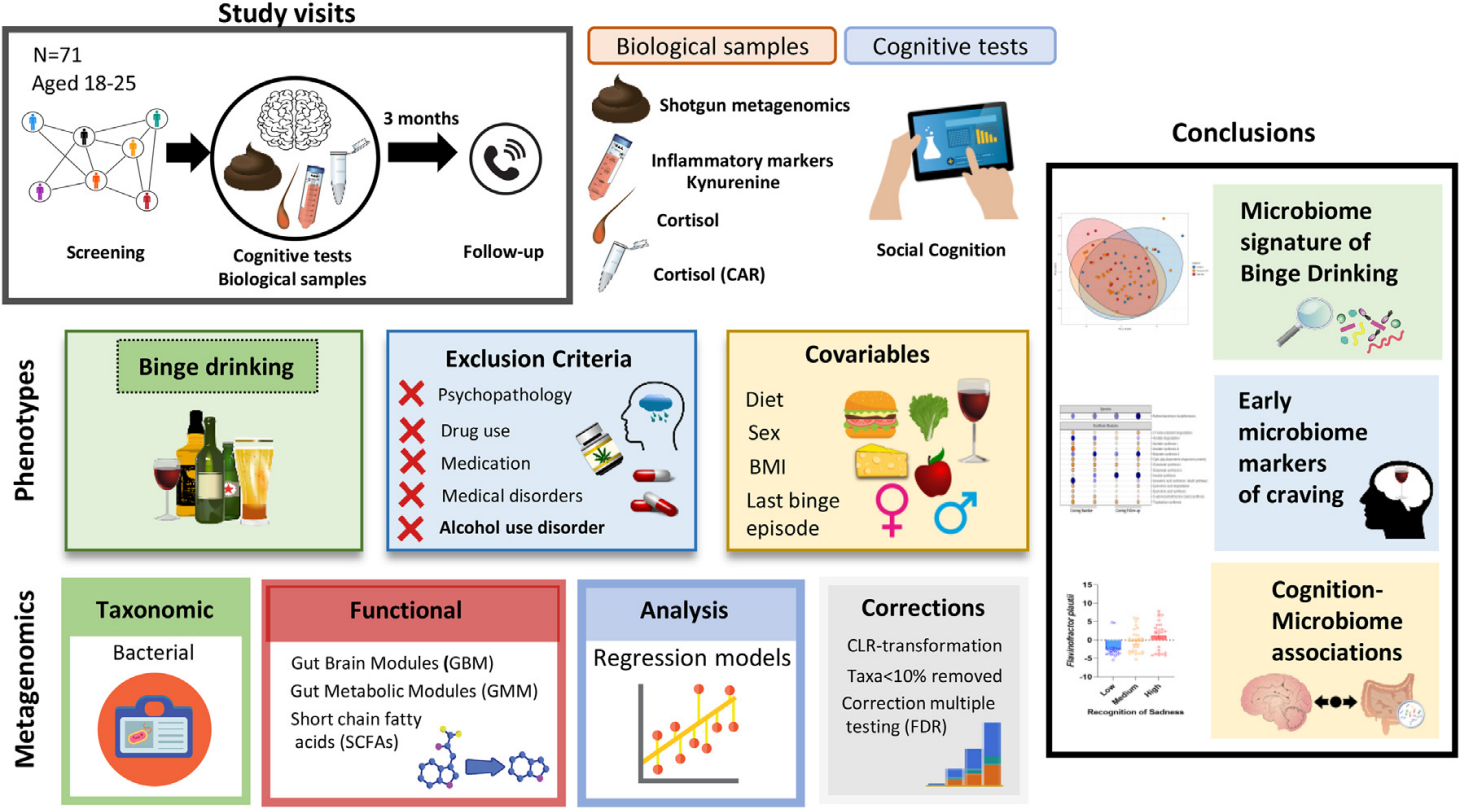
**Study design and outline.** Young binge and non-binge drinkers (N = 71) aged 18–25 were recruited. The study comprised three sessions, stool, blood and saliva samples were collected and cognitive performance was assessed. Craving levels were recorded again three months later (N = 56). Multiple regression models were used incorporating relevant covariables such as diet and correcting for multiple comparisons. Strict inclusion criteria were applied including not having an alcohol use disorder, other drug use or current medication. Findings show binge drinking is associated with a specific microbiome signature and suggest a role of the gut microbiome in regulating social cognition and craving. Biological samples provided differed (saliva N = 64; blood N = 56; hair N = 61).

A total of 71 participants (37 females/34 males) completed the study and 59 (32 females/27 males) participated in the follow-up (see Fig. 1, study design and outline). Power estimation was calculated using *pwr* package from R software (*α* = 0.05, power = 0.9, medium effect size = 0.39, effect type = *f* linear regression by predictor variable). Participants were excluded if they had a significant acute or chronic co-existing illness (including functional gastrointestinal disorders, inflammatory bowel disease, coeliac disease, lactose intolerance etc.), immunological, psychiatric, neurodevelopmental disorders and metabolic disorders (including type I or II diabetes). Furthermore, participants were screened for psychiatric disorders using the International Neuropsychiatric Interview (MINI) at the screening visit. Participants were also excluded if they were currently taking any medication (anxiolytics, pain relievers etc.), with the exception of contraceptives. Participants were not included if they had taken any prebiotic or probiotic supplements or antibiotics 4 weeks prior to enrolment in the study. Participants should have a wash-out period of at least 4 weeks.

Participants were excluded if they scored ≥20 in the Alcohol Use Disorders Identification Test (AUDIT),^30^ as a classical cut-off for probable AUD. Lifetime abstinence from alcohol or a diagnosis of AUD were considered exclusion criteria. Participants were excluded if they had family history of alcoholism in first degree relatives, in this case the Family Tree Questionnaire (FTQ) for assessing family history of alcohol problems was used.^31^ Furthermore, participants having a frequent cannabis use (defined as a frequency of >3 times a month in the last 6 months), having other drug use greater than experimental use (i.e., more than twice per year), and being an habitual smoker (daily smoking) were also excluded. In order to register this, the Drug History Questionnaire was employed.^32^ Participants who were not fluent in English, were colour blind or had dyslexia or dyscalculia were not included.

### Samples and materials

#### Alcohol consumption patterns

Alcohol consumption patterns were measured with the AUDIT and the Alcohol Timeline Follow-back (TLFB). The AUDIT^30^ is a 10-item screening tool developed by the World Health Organization (WHO) to assess alcohol consumption, drinking behaviours, and alcohol-related problems. The alcohol TLFB^33^ is a drinking assessment method that obtains estimates of daily drinking by using a calendar. Individuals provide retrospective estimates of their daily drinking over a specified time period. In this case, the period was the previous 30 days (i.e., specifically 32 days as the 2 days before the evaluation participants were require to not binge drink). Number of binge drinking episodes in the last 30 days, maximum number of drinks (measured as standard drinks) within a single drinking episode, and total drinks (standard drinks) over the past 30 days were the main variables recorded. The self-report instruments employed are gold standard instruments in alcohol research with demonstrated validity, reliability and strong psychometric properties that can provide reliable estimates of general drinking patterns (e.g., binge drinking *versus* non binge drinking).

#### Craving

The Alcohol Craving Questionnaire-Short Form (ACQ-SF-R) was used to measure craving. This version contains 12-items to assess craving for alcohol among alcohol users at the present moment. It assesses the multidimensional aspects of craving: compulsivity (urges and desires in anticipation of loss of control over drinking); expectancy (urges and desires to drink in anticipation of the positive benefits of drinking); purposefulness (urges and desires coupled with intent and planning to drink); emotionality (urges and desires to drink in anticipation of relief from withdrawal/negative affect). At follow-up, the ACQ-SF-R was used together with the Approach and Avoidance of Alcohol Questionnaire (AAAQ)^34^ to capture craving over an extended period of time (i.e., the past week). It records three dimensions: Inclined/Indulgent, Obsessed/Compelled, Resolved/Regulated reflecting mild approach inclinations, intense approach inclinations, and avoidance inclinations, respectively.

#### Other health-related data

Habitual dietary intake was measured using a validated Food Frequency Questionnaire (FFQ).^35^ A detailed description of nutrients and food group values over the past month were obtained, analysed as previously described.^36^ Food groups were included as a covariable in microbiome analysis (full models in excel tables with information on relevant dietary associations can be found in Supplementary Material). Participants rate the frequency of consumption of each food/beverage type on a scale from never or less than once per month to ≥6 per day. Several questionnaires were used to assess psychopathological symptoms: Beck's Depression Inventory (BDI)^37^ and State-Trait Anxiety Inventory (STAI).^38^ Additionally, the Bristol Stool Chart was administered, delivery mode (i.e., vaginal birth or caesarean) was reported and body mass index (BMI) was calculated.

#### Neuropsychological tests and impulsivity questionnaires

##### Emotional processing

We used the Emotion Recognition Task (ERT) and the Affective Go/No-go (AGN) task from the Cambridge Neuropsychological Test Automated Battery (CANTAB). The ERT measures the ability to identify six basic emotions in facial expressions along a continuum of expression magnitude. Computer-morphed images derived from the facial features of real individuals, each showing a specific emotion, are displayed on the screen, one at a time. Each face is displayed for 200 ms and then immediately covered up to prevent residual processing of the image. The participant must select which emotion the face displayed from 6 options (sadness, happiness, fear, anger, disgust or surprise). Outcome measures assessed were number of correct responses and frequency of response per emotion category. The AGN assesses affective biases for positive and negative stimuli. The test consists of several blocks, each of which presents a series of words from two of three different affective categories: Positive (for example, joyful); Negative (for example, hopeless); Neutral (for example, element). The participant is given a target category and is asked to select a word when it matches this category. Key outcome measures where affective bias, valence-specific latency, omission and commission errors.

##### Impulsivity

The Barratt Impulsiveness Scale (BIS-11)^39^ was used. The BIS is a questionnaire designed to assess the personality/behavioural construct of impulsiveness including motor (acting without thinking), attentional (an inability to focus attention or concentrate) and non-planning (lack of forethought).

#### Blood samples

##### Inflammatory markers

Whole blood was collected into 4-mL lithium-heparin containing tubes (Greiner Bio-One, 454029). For the whole blood stimulation, whole blood was diluted 1:10 with Dulbecco's Modified Eagle's Medium/Nutrient Mixture F-12 Ham (Sigma-Aldrich) supplemented with 10% foetal calf serum and 5% penicillin streptomycin. Of the diluted mixture, 500 μL was aliquoted to a 24-well plate. Each well was stimulated with 5 μL of the stimulant *Escherichia* *coli K12* lipopolysaccharide (LPS) which is a Toll-like receptor 4 (TLR4) agonist. Dysregulation of signalling pathways associated with TLR4 receptors is one of the most replicated findings in relation to alcohol and inflammatory processes.^40^ The blood was harvested after 24 h of incubation at 37 °C and stored at −80 °C for later analysis. The following cytokines were analysed both in unstimulated and TLR4 agonist-stimulated conditions: Tumour Necrosis Factor α (TNF-α), Interferon-γ (IFN-γ) and Interleukins (IL-1β, IL-2, IL-10, IL-6, IL-8). Cytokine levels were quantified using the V-PLEX Proinflammatory Panel 1 (human) Kit (MSD, K15049D). Cytokine quantification was done according to the manufacturer's guidelines with one modification, where a total of 100 μL (50 μL in duplicate) sample was added directly onto the plate without dilution (applied for both stimulated and unstimulated conditions).

##### Kynurenine and tryptophan analyses

Tryptophan and kynurenine pathway metabolites were determined as previously described.^41^ Plasma samples were collected into 3-mL K3EDTA tubes (Cruinn Diagnostics Limited, Dublin). Plasma samples were centrifuged at 1500 g for 10 min. The supernatant was aliquoted and stored at −80 °C for later analysis.

#### Cortisol markers

Hypothalamus-pituitary-adrenal axis (HPA axis) function was determined by measuring the concentration of the cortisol awakening response (CAR) in saliva and long-term accumulated cortisol levels in hair, as previously described.^42^ Participants provided four saliva samples from the morning of the day of the evaluation using Salivette tubes (Sarstedt, Germany). The first sample was collected upon wakening, the second one 30 min later, and the third and fourth 45 min and 60 min respectively after wakening. Saliva samples were centrifuged at 1500 g for 5 min, aliquoted, and stored at −80 °C for later analysis.

A hair sample of approximately 150 strands (ideally 2–6 cm long) was cut close to the scalp from the back of the head in a position deemed least noticeable for the participant. The sample was stored at room temperature for subsequent analysis.

Cortisol concentrations were measured in the saliva samples (diluted 1:3, 200 μL of assay buffer and 100 μL of saliva) and in the supernatant resulting from hair samples using enzyme-linked immunosorbent assay (ELISA) kits (Enzo Life Sciences, Exeter, UK) following manufacturer's instruction. The lower limit of detection was 0.919 nmol/L. Area under the curve with respect to ground (AUCg) and to increase (AUCi) were calculated for the cortisol concentrations in the saliva samples.

#### Stool samples

Faecal samples were collected into plastic containers containing an AnaeroGen sachet (Oxoid AGS AnaeroGen Compact, Fischer Scientific, Dublin), and kept cool in a refrigerator until delivery to the laboratory. The sample was aliquoted and stored at −80° for later analysis. Participants were instructed to collect the faecal sample as close to the study visit as possible. DNA was extracted from 250 mg faecal sample using a previously described protocol, which combined the repeat bead beating method with the QIAamp Fast DNA Stool Mini Kit (Qiagen, Germany).^43^ DNA was quantified using the QubitTM 3.0 Fluorometer (Bio-Sciences Dublin, Ireland or Life Technologies or Thermo Fisher Scientific) and the Qubit® dsDNA HS Assay Kit (Bio-Sciences Dublin, Ireland or Life Technologies or Thermo Fisher Scientific), along with being run on a 1.5% agarose gel to check the DNA quality. Extracted DNA was then stored at −20 °C until prepared for shotgun sequencing.

##### Shotgun sequencing library preparation

Whole genome shotgun sequencing library was performed with the Nextera XT kit and the library was prepared according to the Nextera XT DNA Library Preparation Guide (Illumina). Briefly, DNA concentrations were diluted to 0.2 ng/μL and fragmented by incubating at 55 °C for 7 min. Paired-end Nextera XT indexes were added and 12 cycles of amplification process were completed. Samples were purified with AMPure XP beads according to the manufacturer's instructions. In order to calculate molarity, DNA concentration was quantified using the Qubit dsDNA High Sensitivity Assay and amplicon size was measured by Agilent Bioanalyser 2100. Lastly, 1 mM of libraries were pooled before loading on the Illumina NextSeq platform for 150 bp paired-end sequencing.

##### SCFAs

The concentration of SCFAs was analysed by gas chromatography flame ionisation detection (GC-FID) using a Varian 3800 GC system, fitted with a guard column (Restek) connected to an Agilent DB-FFAP column (30 mL × 0.3 mmID × 0.25 μm df) and a flame ionisation detector with a CP-8400 auto-sampler.

### Statistical analysis

Statistical analyses for microbiome data were carried out in R Software (version 4.2.0). Graphs were performed using both R Software and Graphpad Prism (version 8, La Jolla, CA, USA). Complementary analyses were performed using SPSS Statistics Software (version 27, Chicago, IL, USA). A regression approach was followed to better capture the continuity/severity of the drinking pattern and -importantly- to build stronger statistical models incorporating crucial confounding variables for the microbiome analysis such as diet (i.e., food groups from the FFQ). Alcohol variables, more specifically, total drinks, maximum drinks and number of binge drinking episodes, were entered into the multivariate model as independent variables. The models were adjusted by potential confounders. For neuropsychological outputs stepwise multiple regression models were built and multicollinearity was assessed using variance inflation factor (VIF), following a conservative criterion (VIF <3). Descriptive statistics were explored by Pearson's two-tailed and Spearman correlations.

### Taxonomic and functional analysis

Reporting guidelines for human microbiome research (STORMS) was followed^44^ and included in Supplementary Material together with the STROBE list. We performed quality checks on raw sequences from all faecal samples using FastQC. Shotgun metagenomic sequencing data were then processed through analysis workflow that utilizes the Huttenhower Biobakery pipeline,^45^ including Kneaddata, MetaPhlAn3^46^ and HUMAnN3^47^ to obtain species, genes and pathways abundance matrix. Briefly, quality filtering and host genome decontamination (human) was performed using Trimmomatic^48^ and Bowtie 2^49^ via Kneaddata wrapper program with following parameters: ILLUMINACLIP:/NexteraPE-PE.fa:2:30:10, SLIDINGWINDOW:5:25, MINLEN:60, LEADING:3, TRAILING:3. Taxonomic and functional profiling of the microbial community was performed using MetaPhlan3 and HUMAnN3 using default parameters. Next, gene abundance matrix was further collapsed by the KEGG Orthology (KO) term mapping via “humann_regroup_table” function provided within HUMANn3. Gut-brain modules and gut-metabolic modules were calculated using the R version of the Gomixer tool.^50^ Gut-brain modules refers to the presence and abundance of microbial genes that encode for molecules that are known to be neuroactive,^50^ with potential implications in various mental health conditions.^51^

### Bioinformatic analysis

Further data-handling was undertaken in Rstudio GUI (version 2022.2.2.485). In all microbiome analysis with the exception of alpha diversity, taxa with a prevalence of <10% of samples at the species level were excluded from analysis as ratios are invariant to subsetting and this study employs compositional data analysis techniques.^52^

Principal component analysis was performed on centred log-ratio transformed (clr) values using the ALDEx2 library.^53^ The number of permutations was always set to 1000. Beta diversity was computed in terms of Aitchison distance, or Euclidean distance between clr-transformed data. Alpha diversity was computed using the iNEXT library.^54^ CLR-transformed species counts were related to drinking variables using the base R *lm()* function (linear models), adjusting for BMI, dietary intake (i.e., food groups FFQ) and the number of days since the last binge drinking episode. Significant models were selected based on the Benjamini-Hochberg procedure with a q-value of 0.2 as a threshold as well as the confidence interval not containing 0. This procedure controls the False Discovery Rate (FDR) using sequential modified Bonferroni correction for multiple testing. Custom R scripts and functions are available online at https://github.com/thomazbastiaanssen/Tjazi. Regression models were visualized in graphs by showing main effects (β) represented in red (increased) or blue (reduced) with higher colour intensity representing a bigger effect.^47^ Opaque points represent effects that pass FDR.

As a complementary straightforward visualization strategy, drinking tertiles were created a posteriori to depict the characteristics of low drinkers (up to the 25th percentiles), BDs, and high BDs (75th percentile up) individuals departing from the drinking variable that showed the strongest association with microbiome alterations in the regression models.

## Results

### Sample characteristics

**Fig. 2:**
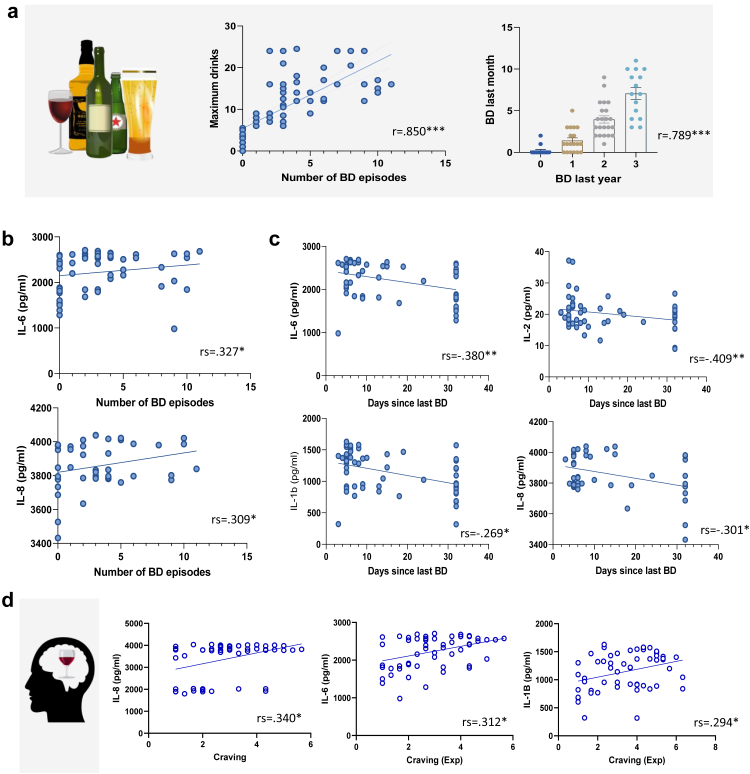
**Inflammatory markers associations for binge drinking (BD) and craving.a.Alcohol consumption data.** Raw drinking scores showing the high correlation between BD episodes (i.e., 60 g or more of pure alcohol on one occasion) in the last month (measured by the TLFB), with maximum drinks and BD in the past year (measured by AUDIT question 3 in which items 2–3 indicate monthly and weekly BD respectively). **b. Binge drinking and inflammatory markers.** BD correlated positively with higher stimulated inflammatory markers. **c. Days since the last binge drinking episode and inflammatory markers.** Significant negative correlations were found for number of days since last BD episode and stimulated inflammatory markers, i.e., the more recent the BD episode, the higher the inflammation. **d. Craving and inflammatory markers.** Higher craving (expectancy dimension) correlated with higher responsiveness of stimulated inflammatory markers.

Sociodemographic and clinical variables are shown in Table 1. No gender differences were found for number of binge drinking episodes. However, males consumed a higher number of maximum drinks within a drinking episode (t (57.03) = −2.810, p < .007). Therefore, sex was included as a covariable in the multivariate models. Raw data describing number of binge drinking episodes, number of drinks per session, and total drinks in the last month is represented in Fig. 2a.

**Table 1:** Sociodemographic and clinical variables.

	Total Sample		Males		Females	
	N = 71		N = 34		N = 37	
	Mean (SD)	Min-Max	Mean (SD)	Min-Max	Mean (SD)	Min-Max
Age	22.05 (1.86)	18.85–25.99	21.86 (1.81)	18.85–25.99	22.22 (1.92)	18.85–25.70
AUDIT Total	8.54 (5.57)	0–19	9.53 (5.41)	0–19	7.62 (5.63)	0–19
AUDIT-C^a^	5.03 (2.47)	0–10	5.53 (5.41)	0–10	4.57 (2.44)	0–9
Number of BD episodes^b^	3.14 (3.09)	0–11	3.5 (2.8)	0–9	2.81 (3.34)	0–11
Number of days since last BD	14.68 (11.32)	3–32	13.68 (11.05)	3–32	15.59 (11.63)	3–32
Maximum drinks^c^	10.47 (6.89)	0–24.5	12.79 (7.77)	0–24.5	8.34 (5.22)	0–21
Total drinks	42.09 (37.57)	0–143.5	48.82 (37.05)	0–127.5	35.9 (37.47)	0–143.5
BDI	5.68 (4.99)	0–19	6.97 (4.86)	0–17	4.50 (4.89)	0–19
STAI (trait)	28.32 (5.98)	20–45	27.94 (5.46)	20–41	28.65 (6.46)	20–45

a First three items of AUDIT.

b TLFB calendar of one month (32 days) previous to the evaluation.

c Maximum drinks is defined as maximum standard drinks (SDs) withing a drinking episode.

### Inflammatory markers, kynurenine markers and cortisol

Greater responsiveness to TLR4 stimulation was associated with higher binge drinking. In TRL4 agonist-stimulated blood, number of binge drinking episodes correlated positively with IL-6 and IL-8 (Spearman's correlation coefficient [rs] = .327, p = .014; rs = .309, p = .020), see Fig. 2b. Interestingly, a significant negative correlation was found for number of days since last binge drinking episode for IL-6 (rs = −.380, p = .004), for IL-8 (rs = −.301, p = .024); and IL-1β (r = −.409, p = .002); IL-2 (r = −.269, p = .045) (Fig. 2c). No correlations were found for cytokine markers in unstimulated conditions, as well as for any kynurenine and cortisol markers. In summary, while no association were found unstimulated conditions, binge drinking was associated with greater responsiveness in stimulated conditions.

### Craving

As expected, total craving (ACQ) correlated positively with number of binge drinking episodes (r = .402, p = .001). Craving showed positive correlations with stimulated inflammatory markers IL-6, IL-8 and IL-1β. Craving correlated with stimulated IL-6, IL-8 and IL-1β (rs = .312, p = .019; rs = .340, p = .010; r = .294, p = .028- expectancy dimension), see Fig. 2d. In summary, craving was associated with higher responsiveness of the immune system.

### Social cognition and impulsivity

**Fig. 3:**
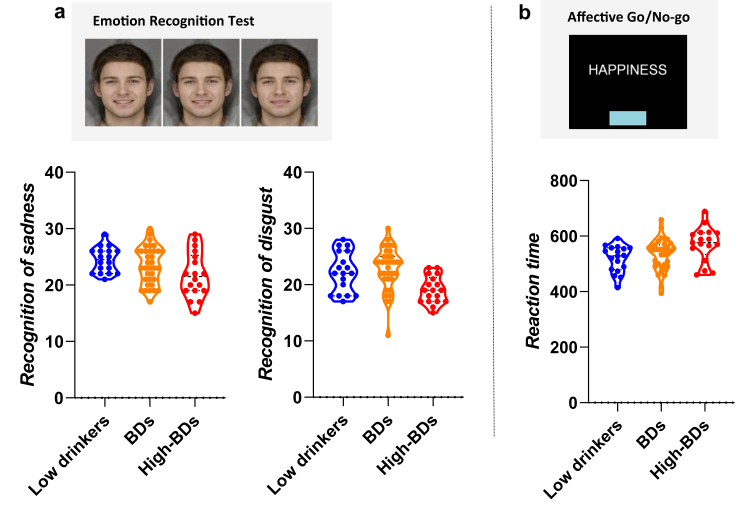
**Neuropsychological performance in social cognition and binge drinking(BD).a. Emotional functioning and binge drinking: raw data divided by tertiles.** Higher BD was associated to poor emotion recognition for sadness and disgust (lower correct responses) in the Emotional Recognition Task (ERT). The Tertiles visualization depicts raw data. Statistical models with regression coefficients are described in text and full models can be found in Supplementary Material. **b. Affective bias and binge drinking: raw data divided by tertiles.** Higher BD was associated to slower reaction time (measured as higher latencies for correct responses) in the Affective Go/No-go (AGN). The Tertiles visualization depicts raw data. Statistical models with regression coefficients are described in text and Supplementary Material.

Means and standard deviations for neuropsychological performance are shown in Table 2. In the multivariate models, gender and psychopathological symptoms were included as potential confounders, as recommended by previous research.^19^ Full model coefficients are detailed in Supplementary Material Table S1. As a straightforward way to facilitate visualization of results, Fig. 3 depicts main results by drinking tertiles (low drinkers, BDs and high BDs).

**Table 2:** Means and standard deviation for neuropsychological performance on social cognition.

	Mean (SD)
	N (71)
**ERT**	
Number of correct responses	129.06 (9.70)
Number correct happiness	26.89 (2.77)
Number correct sadness	23.29 (3.37)
Number correct fear	15.18 (5.53)
Number correct surprise	23.61 (2.68)
Number correct disgust	21.73 (3.75)
Number correct anger	18.53 (2.61)
**AGN**	
Emotional bias	−8.73 (31.80)
Total latency correct	522.33 (56.31)
Commissions	17.62 (9.82)
Omissions	12.03 (9.46)

ERT = Emotional Recognition Test (ERT), AGN = Affective Go/No-go.

Higher number of drinks within a drinking episode predicted lower recognition of sadness (F(1, 67) = 5.16, p = .026, R^2^_adj_ (Adjusted R-squared) = .06, β = −.269) and lower recognition for disgust (F(1, 67) = 10.49, p = .002, R^2^_adj_ = .12, β = −.370), see Fig. 3a. Secondly, regarding the AGN, no effects were found for emotional bias. Total drinks was the best predictor of greater latency (slower reaction time) for correct responses (F(1, 69) = 15.806, p < .001, R^2^_adj_ = .183, β = .442), see Fig. 3b. Results did not vary by shift or non-shift condition or valence-specific latency. No associations were found for commission or omission errors.

Finally, as expected, impulsivity correlated positively with higher number of binge drinking episodes (Attentional, Pearson correlation coefficient [r] = .310, p = .009; Motor, r = .263, p = .028; Non-Planning, r = .253, p = .035). In summary, binge drinking was associated with lower performance facial emotion recognition and higher impulsivity.

### Microbiome data

#### Microbiome and alcohol

No differences were found in relation to gender and delivery mode. BMI, diet and the day of the last binge drinking episode were maintained as significant covariables in the models, see Supplementary Material Tables S2, S4, S5 for full model's coefficients. In order to facilitate the visualization of the results, drinking tertiles (low drinkers, BDs and high BDs) -based on maximum drinks were computed.

**Fig. 4:**
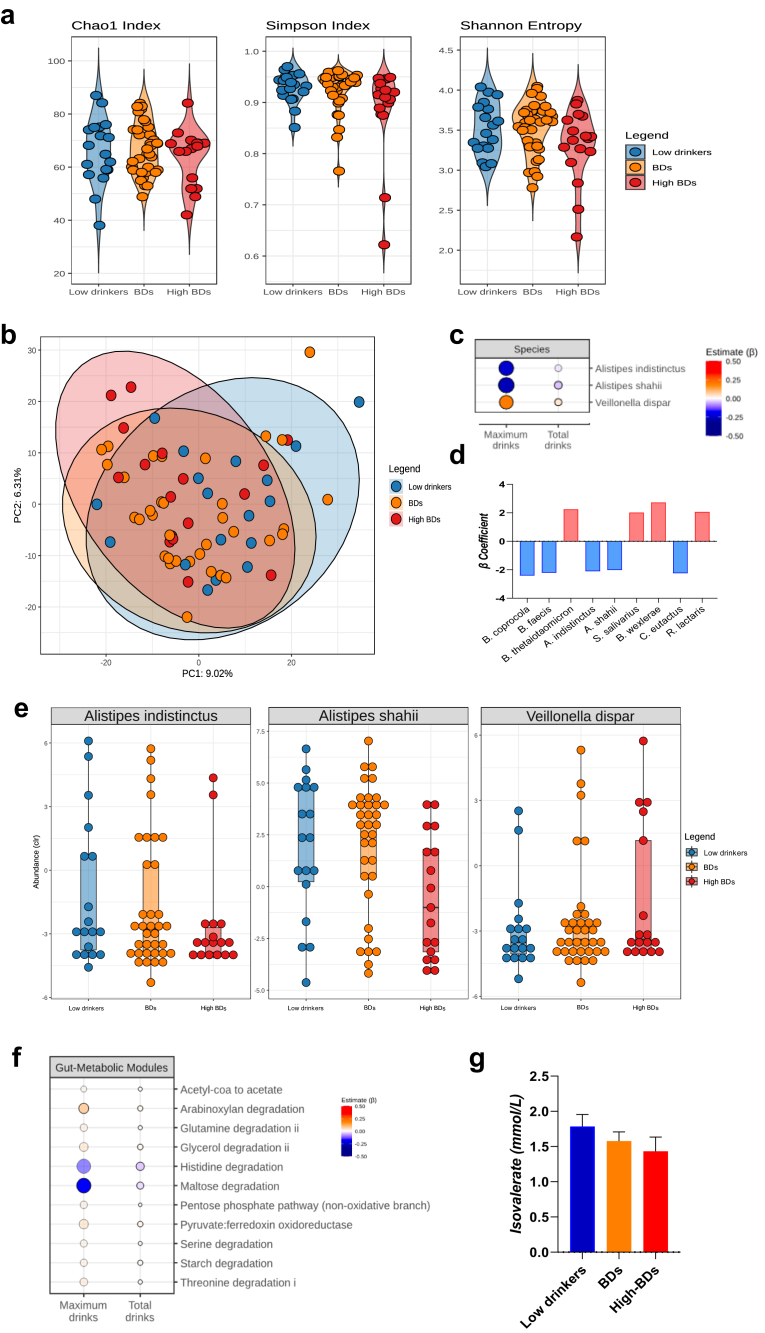
**Microbiome alterations in young binge drinkers.a. Alpha diversity by drinking tertiles.** Alpha diversity was computed using the iNEXT library. Alpha diversity was not associated with BD variables. **b. Beta diversity by drinking tertiles.** Beta diversity was computed in terms of Aitchison distance, or Euclidean distance between clr-transformed data. Beta diversity changes were linked to maximum drinks per session (PERMANOVA [p = 0.007, R2 = 0.023]), depicted here as a principal component analysis (PCA) graph. **c. Gut microbiome composition and binge drinking.** Regression models (l*m()* function) of CLR-transformed species counts were related to drinking variables, adjusting for BMI, dietary intake and the number of days since the last BD episode. False Discovery Rate (FDR) using sequential modified Bonferroni correction for multiple testing was applied. Compositional results (revealed alterations in some species of the genus *Alistipes* (reductions) and *Veillonella* (increases) linked to higher number of drinks per occasion. The effect (β) is represented in red (increased) or blue (reduced) with higher colour intensity representing a bigger effect. Opaque points represent effects that pass FDR. **d. Recency of binge dsrinking episodes and gut microbiome composition.** A recent BD episode (the covariable days since last BD episode) was associated with further widespread microbiome alterations such as *Bacteroides spp., Alistipes spp., Blautia wexlerae, Ruminococcus lactaris, Coprococcus euctactus* among others. **e. Gut microbiome composition by drinking tertiles.** Compositional results are shown by drinking tertiles (low drinkers, BDs and high BDs) based on maximum drinks, in order to facilitate visualization of regression models depicted in c. The Tertiles visualization depicts raw data. Statistical models with regression coefficients are described in text (and in c and f) and full models can be found in Supplementary Material. **f. Gut-metabolic modules and binge drinking.** Gut-metabolic modules (R Gomixer tool) showed reduced histidine and maltose degradation linked to higher BD. **g. Short-chain fatty acids (SCFAs) and binge drinking.** Higher BD (higher number of drinks per session) was associated with lower isovalerate.

Alcohol-related alterations in microbiome composition are shown in Fig. 4. First, in terms of microbiome richness, no differences were found for alpha diversity indeces (Fig. 4a). Subtle beta diversity differences were linked to higher number of drinks per session (PERMANOVA [p = .007, R-squared [R2] = .023], depicted in Fig. 4b as a principal component analysis [PCA]), further description can be found in Supplementary Material. Compositional results revealed alterations in some species of the genus *Alistipes* (reductions) and *Veillonella* (increases) (Fig. 4c and d). Additionally, a recent binge drinking episode was associated with further widespread microbiome alterations (Fig. 4d), including *Bacteroides* *spp.*, *Alistipes spp., Blautia wexlerae*, *Ruminococcus lactaris, Coprococcus euctactus,* among others (for full models Supplementary Table S2). No associations were found for gut-brain modules. Gut-metabolic modules showed alcohol-related alterations, particularly reduced histidine and maltose degradation, see Fig. 4f. Finally, no correlations were found for alcohol variables with the Bristol Stool Chart. In summary, binge drinking was associated with compositional and functional gut microbiome alterations.

#### Short Chain Fatty Acids (SCFAs) and alcohol

Higher number of drinks per session was associated with lower isovalerate (F(1, 67) = 5.52, p = .022, R^2^_adj_ = .063, β = −.278), see Fig. 4g. Full models can be found in Supplementary Material Table S3, diet, (days since) last binge drinking episode and BMI were included as covariables.

#### Microbiome and craving

**Fig. 5:**
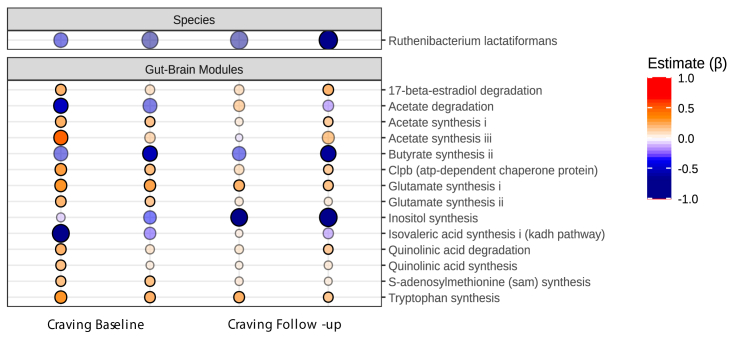
**Gut microbiome and craving.** Regression models (lm() function) of CLR-transformed species counts were related to craving variables, adjusting for BMI, dietary intake and the number of days since the last BD episode. False Discovery Rate (FDR) using sequential modified Bonferroni correction for multiple testing was applied. The effect (β) is represented in red (increased) or blue (reduced) with higher colour intensity representing a bigger effect. Opaque points represent effects that pass FDR. Craving was associated with reductions in the *Ruthenibacterium lactiformas* species, both at baseline and at follow-up (3 months later). Gut-brain modules (the potential of the microbiome to produce chemicals with neuroactive potential) was calculated with R Gomixer tool. A number of gut-brain modules showed associations with craving, such as reduced butyrate and inositol synthesis and increased acetate, glutamate and tryptophan synthesis. Associations were found for craving at baseline and at follow-up (3 months later). Baseline craving dimensions showing significant associations were expectancy and purposefulness (first and second column respectively) and at follow-up obsessed and regulated dimensions (third and fourth column).

Associations between microbiome and craving are depicted in Fig. 5. After controlling for confounders, greater craving was associated with reductions in *Ruthenibacterium lactiformans*, both at baseline and at follow-up (3 months later). A number of gut-brain modules showed craving-related associations. Particularly, reduced butyrate and inositol synthesis and increased acetate, glutamate and tryptophan synthesis were associated with higher craving. Gut-metabolic modules displayed numerous alterations associated with higher craving including reduced acetate degradation and higher acetate synthesis, reduced butyrate production, reduced valine and cysteine degradation were linked to higher craving, while increased acetyl-CoA to acetate and ethanol production showed a positive association with craving at baseline and follow-up. Full models can be found in Supplementary Material Table S4, diet, (days since) last binge drinking episode and BMI were included as covariables. Results for gut-metabolic modules and craving can be found in Supplementary Material Fig. S1. In summary, craving was associated with compositional and functional alterations in the gut microbiome across time.

#### Microbiome, social cognition and impulsivity

**Fig. 6:**
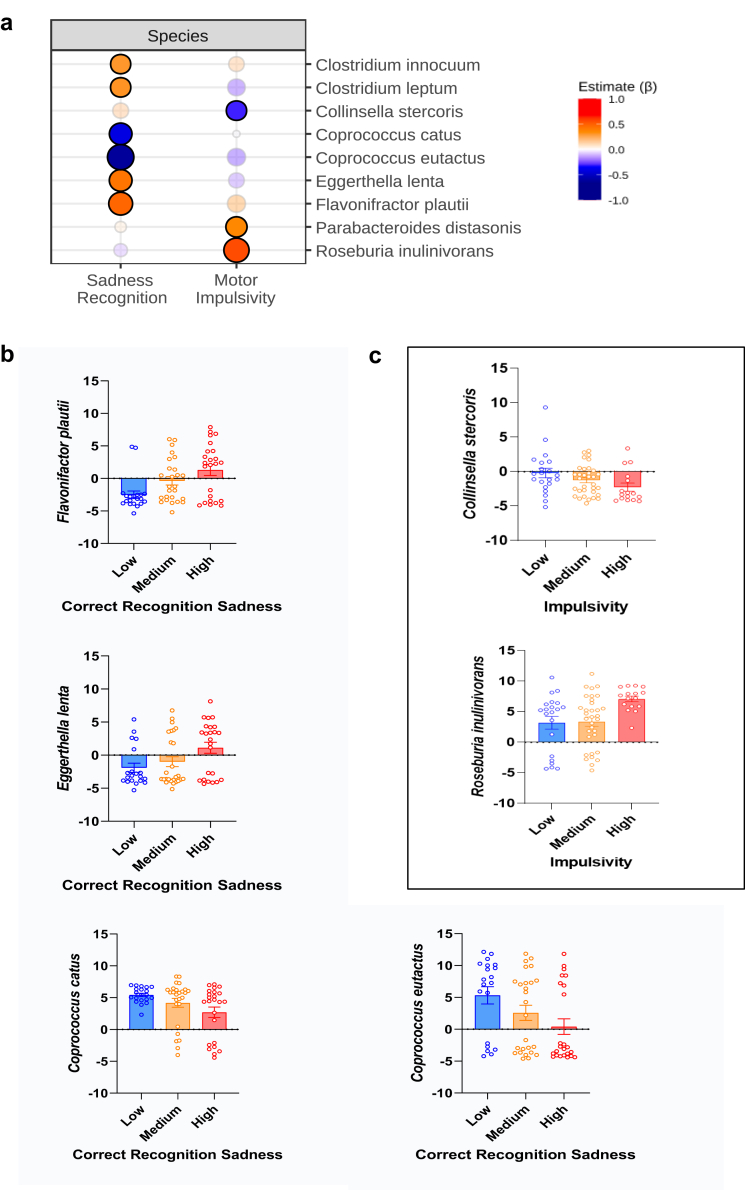
**Microbiome associations with social cognition and impulsivity.a. Gut microbiome composition and emotional functioning and impulsivity.** Regression models (lm () function) of CLR-transformed species counts were related to social cognition difficulties and impulsivity, adjusting for BMI, dietary intake and the number of days since the last BD episode. False Discovery Rate (FDR) using sequential modified Bonferroni correction for multiple testing was applied. The effect (β) is represented in red (increased) or blue (reduced) with higher colour intensity representing a bigger effect. Opaque points represent effects that pass FDR. Emotion recognition showed a strong association with microbiome composition. Particularly, a decrease in *Clostridium spp., Flavonifractor plautii, Eggerthella lenta* and an increase of *Coprococcus spp.* was associated with poor recognition of sadness. Higher motor impulsivity was associated with several species, e.g., reduction of *Collinsella*, increased *Roseburia* and *Parabacteroidetes*. **b and c. Gut microbiome and cognitive performance and impulsivity by drinking tertiles.** Previous results are visualized by tertiles depending on emotion recognition performance and impulsivity, respectively. Tertiles represent raw data as a complementary visualization strategy.

Associations between microbiome, emotional processing and impulsivity are depicted in Fig. 6. Emotion recognition showed a strong association with several species. Particularly, a decrease in *Clostridium spp.*, *Flavonifractor plautii*, *Eggerthella lenta* and an increase of *Coprococcus spp.* Was associated with poorer recognition of sadness (facial emotion recognition) in the ERT (see Fig. 6a). No associations were found for cognitive performance in the AGN task. Finally, higher impulsivity was associated with several species, e.g., reduction of *Collinsella spp.*, increased *Roseburia* and *Parabacteroidetes spp.* (Fig. 6b and c, represent previous results by tertiles depending on cognitive performance and impulsivity). Regarding potential gut-brain axis mediators, microbiome alterations were associated with higher responsiveness in TLR4 stimulated inflammatory markers. *Coprococcus eutactus* (linked to poor facial emotion recognition for sadness) showed a partial positive correlation with IL-6 (r = .544, p = .045). In summary, strong links between gut microbiome composition and emotional functioning and impulsivity were observed.

## Discussion

There is a growing appreciation that the microbiome may play a role in shaping brain and behavioural processes at key windows across the lifespan. This study identifies gut microbiome alterations in BDs and its cognitive correlates during adolescence. These findings are of importance in order to determine early biomarkers of alcohol misuse before an addiction develops. Additionally, we investigated changes in potential mediators of gut-brain communication such as the immune response (baseline and stimulated blood cytokine response), SCFAs, the kynurenine pathway and the cortisol response.

First, the number of binge drinking episodes correlated with higher responsiveness of stimulated cytokines (IL-6 and IL-8). Additionally, the more recent the binge drinking episode, the greater stimulated blood cytokine response (mainly increases across TLR4 stimulated cytokines IL-6, Il-8, IL-1β). Similarly, craving has been related to higher concentration of inflammatory markers in blood among AUD patients.^55^ Even a single binge drinking episode increased serum endotoxin levels likely due to translocation of gut bacterial products and disturbs innate immune responses that can contribute to the deleterious effects of binge drinking.^56^

As expected, greater number of binge drinking episodes were associated with greater impulsivity, which replicates a well-known association in the binge drinking literature.^57^ Regarding social cognition, higher binge drinking was associated with difficulties in emotion recognition of disgust and sadness in the Emotional Recognition task. Higher binge drinking was linked to slower processing speed in the Affective Go/No-go task. This is in line with previous studies showing cognitive difficulties in BDs including slower reaction times.^4^ Paralleling findings in patients with alcoholism, a growing number of papers show that young BDs appear to have difficulties (to a lower degree) in social cognition,^19^ such as emotional recognition,^58^ affective memory bias^59^ or theory of mind.^60^ Social cognition deficits are of relevance as they have been linked to the development and maintenance of alcohol addiction.^21^ In the light of immature cognitive processes during adolescence (e.g., lower emotional regulation skills or lower affective control^18^) further difficulties in emotional processing could possibly increase the risk psychopathological disorders.

Microbiome models revealed that higher number of drinks within a single drinking episode, was associated with compositional and functionality changes in the gut microbiome. While no alpha diversity differences were observed, significant associations were found for beta diversity. A recent study, also reported beta diversity differences associated with higher blood alcohol concentration (BAC) in young people.^61^ In non-human primates, long-term binge drinking induced changes both in alpha and beta diversity indeces.^7^ At the species level, we observed reductions in some species of the genus *Alistipes* and increased *Veillonella dispar.* Having had a recent binge drinking episode was associated with further widespread microbiome alterations implicating a larger number of genus. Altogether, this could suggest distinct transitory short-term changes and more stable alterations. Decreases in *Alistipes* have been observed in patients with liver disease,^62^ however, the contrary (i.e., increased relative abundances of this genus) have also been reported in animal models of chronic alcohol use.^63^ In particular, decreases in *Alistipes shahii* and *Alistipes indistictus* were observed in patients with cirrhosis,^64^^,^^65^ as well as increases in opportunistic pathogens such as *Veillonella*.^65^, ^66^, ^67^, ^68^ Adolescence, as a neurodevelopmental period with particular sensitivity to external insults. Hypothetically, this could possibly extend into the gut microbiota.^23^ Thus, microbiome and immune alterations could contribute to an *allostatic load* that might increase the risk of addiction vulnerability.^21^

Regarding microbiome metabolic modules, analysis showed reductions in histidine and maltose degradation linked to higher number of drinks per drinking session. Histidine is one of the most studied amino acids in chronic alcohol consumption due to histamine toxicity.^69^ The reduced degradation of histidine may result in increased availability for conversion to histamine associated with higher number of drinks per occasion and may favour a pro-inflammatory state in the intestinal tract.^70^ Histamine is present at relatively high concentrations in the gut, particularly during inflammatory responses.^71^ In addition, a reduction in the degradation of maltose could lead to a lower amount of glucose availability. Decreases in glucose levels have been observed after in a preclinical binge drinking study, forcing the use of other compounds such as acetate for alternative energy sources in the brain.^72^

The analysis of faecal SCFAs revealed a reduction in isovalerate linked to maximum drinks. In a previous study, lower abundance of gut-brain modules related to isovalerate synthesis have been reported in AUD patients,^66^ and isovalerate levels have been shown to be increased with respect to baseline levels in AUD patients after receiving a faecal microbiota transplantation (FMT).^73^ Increases in isovalerate have been linked to bacteria of the genus *Alistipes* among others,^74^ which might also relate to the decrease in the proportion of this taxa in the current study.

Craving was associated with alterations in microbiome composition and neuroactive potential. Reductions in the *Ruthenibacterium lactiformans* species, were linked to higher craving across time (both at baseline and 3 months later). This species belongs to the *Ruminococcaceae* family and could constitute an interesting candidate as an early biomarker of craving. In human studies, decreased *Ruminococcaceae* taxa was observed in AUD patients with high levels of craving.^9^ Interesting results were observed in a recent trial in which FMT administration was performed in AUD subjects with cirrhosis. Those patients that received the FMT enriched with *Lachnospiraceae* and *Ruminococcaceae* (deficient taxa in this population) presented a short-term improvement in inhibitory control and reduced craving that negatively correlate with *Ruminococcaceae* family.^73^

In terms of neuroactive potential, we observed that higher craving was associated with reduced butyrate and inositol synthesis and increased acetate, glutamate and tryptophan synthesis (measured as gut-brain modules, a framework that captures the known microbial pathways for synthesis and degradation of neurochemicals by members of the human gut microbiota^50^). Similarly, in a study with AUD patients, a higher abundance of gut-brain modules related to glutamate synthesis, and a lower abundance related to butyrate, isovalerate, and degradation of glutamate was observed.^66^ Chronic alcohol use has been associated with lower levels of butyrate; a SCFA involved in the maintenance of gut barrier integrity.^75^ *In vitro* experiments reported that butyrate can protect against alcohol-induced toxicity. In this line, preclinical experiments performed in mice showed that butyrate was able to reduce both ethanol intake and preference.^76^ Also, a metagenomic study revealed that alcohol dependence was inversely associated with the levels of butyrate-producing species from the *Clostridiales* order.^77^ Elevated acetate levels have been shown to reprogram gut microbiota during alcohol consumption.^78^ Acetate is the major product of alcohol oxidation with important implications as an energy source in the brain.^79^ Acute alcohol intoxication has been associated with reductions in glucose metabolism in the brain and an increase in acetate uptake, suggesting a persistent reliance on acetate as an alternative brain energy source during alcoholic intoxication.^80^^,^^81^ Alcohol-derived acetate modulates brain function (e.g., by altering neuronal histone acetylation), contributing to the development of addictive behaviours by regulating gene expression.^82^ Therefore, other peripheral sources of physiological acetate (i.e., the gut microbiome) could affect central histone acetylation and brain function in a similar manner.

There is evidence that the gut microbiome is involved in the synthesis and secretion of neurotransmitters -implicated in alcohol addiction^83^- that may have an indirect impact on the brain.^84^^,^^85^ Similarly to our findings, in an animal model of binge drinking, adolescent rats exhibited alterations in caecal neurotransmitters including reductions of glutamate and increases in serotonin.^20^ Inositol, a key metabolic precursor in the phosphatidylinositol pathway, acts as a second messenger system for numerous neurotransmitters (i.e., serotonin) involved in affective disorders. Inositol has shown certain therapeutic effects in the spectrum of disorders responsive to serotonin re-uptake (i.e., depression, panic and obsessive-compulsive disorders), which might explain inositol reductions in the present study being linked to higher craving.^86^^,^^87^

Finally, we observed strong associations between gut microbiome composition and social cognition and impulsivity. Higher motor impulsivity was associated with several species, e.g., reduction of *Collinsella spp.*, increased *Roseburia* and *P.arabacteroidetes spp*. Previous preclinical studies have shown links between microbiome composition and impulsivity in the context of chronic alcohol,^88^ and in an addiction-like phenotype.^89^ Alterations in *Collinsella* have been observed in children with attention deficit hyperactivity disorder (ADHD).^90^ Increased abundance of *Roseburia* have been described in a number of psychiatric disorders that display impulsivity deficits such as ADHD and binge eating.^91^

Poor emotion recognition, particularly difficulties in facial recognition of sadness were associated with reduced abundance of *Clostridium spp., Flavonifractor plautii, Eggerthella lenta* and increased abundance in *Coprococcus spp.* in the current study. In particular, increased abundance of *Coprococcus eutactus* correlated positively with stimulated inflammatory markers (IL-6, i.e., higher responsiveness), which could point to the immune system as a potential mediator in microbiome-emotional functioning alterations. Although more research is needed to clearly elucidate mechanisms, mounting preclinical evidence have demonstrated cognition-microbiome links,^10^^,^^66^^,^^92^ both in the specific context of alcohol use, as well as in other disorders. Some have pointed towards the microbiome being able to cause brain and behavioural alterations.^8^^,^^21^ Gut microbiota and its metabolites appear to play a role in regulating social behaviour and brain development of regions implicated in socio-affective processes including normal HPA axis, hippocampal prefrontal and amygdala development.^12^ A recent study demonstrated a link between microbiome alterations and sociability levels (self-reported questionnaire) in AUD patients.^8^ The authors performed FMT administration from AUD patients to mice and demonstrated that this manipulation was able to induced impaired sociability in mice.^8^ In a preclinical study, microbiome composition was positively associated to AUD severity, and correlated to decreased in dopamine D2 receptor mRNA expression,^88^ which has been implicated in social cognition.^93^ In healthy individuals, gut microbial profiles were associated with emotional processing (e.g., less hippocampal activity viewing negative valences images) in a brain imaging study.^92^ Social cognition and emotional functioning have been further investigated through the use of probiotics, i.e., live bacteria that, when ingested in adequate amounts, produce health benefits.^94^ In different mouse models of autism, a probiotic administration could reverse social behaviour deficits.^15^ In healthy participants, 4-week administration of probiotics was associated with changes in brain activity patterns related to emotional memory and emotional decision-making tasks,^95^ and alterations in brain activity and functional connectivity while performing different emotional tasks.^96^^,^^97^ In stressed adults, a probiotic intervention resulted in faster reaction times in an emotional recognition task and reduced levels of proinflammatory cytokines.^98^ Taken together, these findings appear to support the growing literature that points to the gut microbiome as a regulator of social cognition across species and disorders.^10^^,^^12^

The study is strengthened by using neuropsychological tests (in comparison with previous self-reported scales); incorporating a longitudinal element for craving characterization over time; an in-depth examination of mediators in the gut-brain communication, having strict selection criteria and a deeper level of microbiome analysis (shotgun next-generation sequencing) that allows for better characterization of compositional profile and functional potential. One of the main limitations of the current study is the observational nature, more mechanistic studies are needed to draw causal relationships and unravel the neurobiological underpinnings behind these associations. The lack of blood metabolomic analysis could also be considered as a limitation. Another limitation is that, even when the study focuses on an age range in which binge drinking peaks, the study doesn't include the age range of younger adolescents which would be of interest for future studies. Participants were instructed to abstain from drinking more than two units two days before the second visit, however no tests were performed to confirm the adherence, which could be a potential a limitation of the study. Finally, even when this sample is representative of the young population who drinks alcohol, there might be a slight bias towards a less representative sample in favour of greater control. For example, frequent cannabis use or psychopathology as exclusion criteria could likely bias the sample towards having a population of “super healthy/pure” binge drinkers. This affects the current study and the majority of studies on young drinkers and entails a matter of control over more extensive representativeness. Future studies should consider including a group of young drinkers with comorbid drug use or psychopathology.

In conclusion, this study identifies a gut microbiome signature in young BDs in the absence of addiction. We characterize potential early microbiome markers of craving and show alterations in stimulated inflammatory markers as one of the potential mediators in gut-brain axis communication. We identify strong links between emotional processing and gut microbiome in line with the growing literature on the gut microbiome as a regulator of social cognition. Alterations in the Microbiome-Gut-Brain axis could fuel further dysregulation and contribute to increase the risk of developing future psychopathology, which is of special relevance during the vulnerability period of adolescence.

## Data sharing statement

Raw models are provided in excel tables in supplementary data files. Custom R scripts and functions are available online at https://github.com/thomazbastiaanssen/Tjazi. The datasets used and/or analysed during the current study are available from the corresponding author on reasonable request.

## Declaration of interests

J.F.C has research support from Cremo, 10.13039/100007082Pharmavite, 10.13039/100004352DuPont and Nutricia. He has spoken at meetings sponsored by food and pharmaceutical companies. G.C. has received honoraria from Janssen, Probi, and Apsen as an invited speaker; is in receipt of research funding from 10.13039/100007082Pharmavite, 10.13039/501100003144Fonterra, Nestle and Reckitt; and is a paid consultant for 10.13039/501100012030Yakult, Zentiva and Heel pharmaceuticals. All the authors declare no competing interests.

## Appendix A Supplementary data

Sup Material
