# Supplementary material for: The Microbiome-Gut-Brain axis regulates social cognition & craving in young binge drinkers
Source: eBioMedicine. 2023 Feb 2;89:104442. doi: 10.1016/j.ebiom.2023.104442 (PMC10025767; doi:10.1016/j.ebiom.2023.104442)
Supplement: Sup Material [file mmc1.pdf]

Table 1. Regression coefficients for social cognition and drinking variables.

Predictors	Unstandardized coefficients		Standardized coefficients	*t*	*p*	Model
	B	*SE*	*β*			VD
						**ERT**
Maximum drinks	-.133	.058	-.269	-2.272	.026	Correct responses sadness
Maximum drinks	-.210	.065	-.370	-3.240	.002	Correct responses disgust
						**AGN**
Total drinks	.684	.172	.442	3.976	.001	Total latency for correct responses

Gender and psychopathology (STAI trait and BDI) were included as covariables

Table 3. Regression coefficients for short chain fatty acids and drinking variables.

Predictors	Unstandardized coefficients		Standardized coefficients	*t*	*p*	Model
	B	*SE*	*β*			VD
Coffee	4.037	1.574	.299	2.566	.013	Acetate
High fat dairy products	4.016	1.541	2.97	2.606	.011	Propionate
BMI	.909	.415	.250	2.191	.032	
Processed meats	.535	.218	.290	2.460	.017	Isobutyrate
Grains	4.843	.816	.407	3.789	.001	Butyrate
Maximum drinks	1.278	.247	.354	3.299	.002	
Maximum drinks	-.031	.013	-.278	-2.349	.022	Isovalerate

PERMANOVA

s2(dist.clr ~ MaxDrinks_Tertiles, data = metadata, method = "euclidean", permutations = 1000)

Permutation test for adonis under reduced model

Terms added sequentially (first to last)

Permutation: free

Number of permutations: 1000

adonis2(formula = dist.clr ~ MaxDrinks_Tertiles, data = metadata, permutations = 1000, method = "euclidean")

	Df	SumOfSqs	R2	F	Pr(>F)	
MaxDrinks_Tertiles	1	2897	0.02295	1.5969	0.006993	**
Residual	68	123353	0.97705			
Total	69	126250	1.00000			

Signif. codes: 0 ‘***’ 0.001 ‘**’ 0.01 ‘*’ 0.05 ‘.’ 0.1 ‘ ’ 1

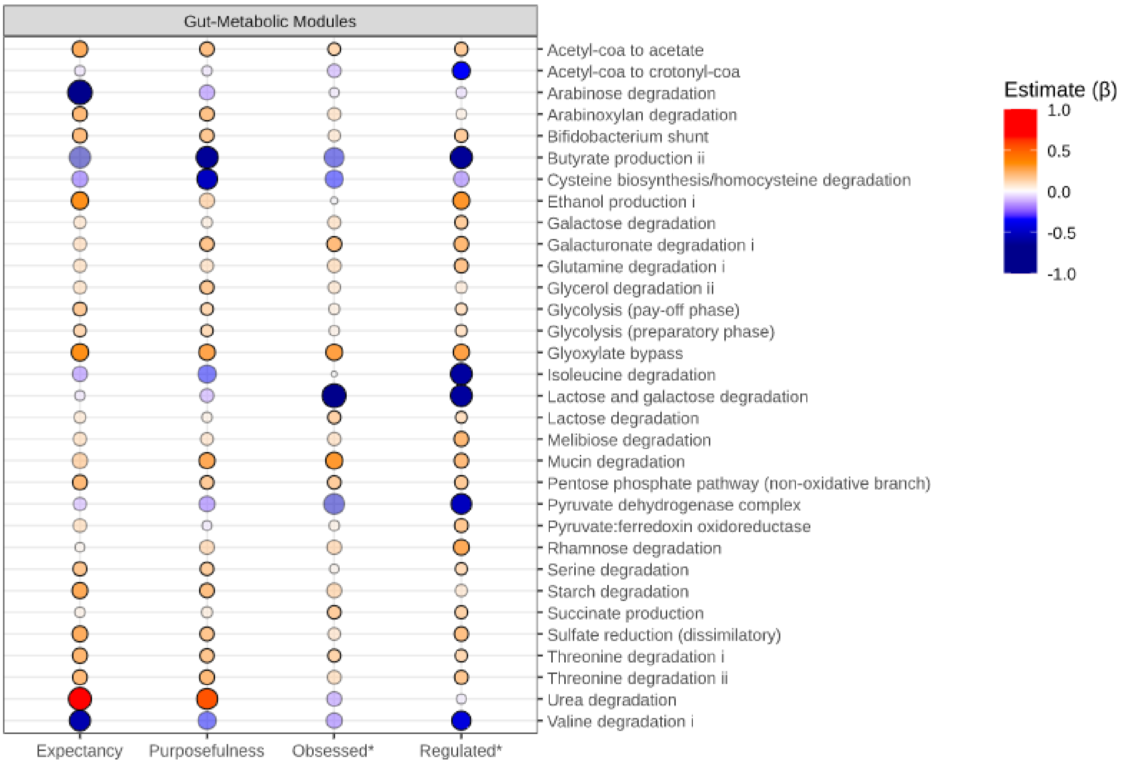

Figure 1. Gut metabolic modules and craving. A number of gut-metabolic modules showed associations with craving, such as reduced butyrate production, cysteine degradation, increased glycoxylate bypass, acetyl-coa to acetate, ethanol production. Craving dimensions at baseline that showed significant associations were expectancy and purposefulness, while obsessed and regulated were significant at follow-up.
